# Synergistic Anti-Tumor Effect of mTOR Inhibitors with Irinotecan on Colon Cancer Cells

**DOI:** 10.3390/cancers11101581

**Published:** 2019-10-17

**Authors:** Damien Reita, Cyril Bour, Radhia Benbrika, Audrey Groh, Erwan Pencreach, Eric Guérin, Dominique Guenot

**Affiliations:** 1 Progression Tumorale et Microenvironnement, Approches Translationnelles et Epidémiologie, EA3430, Université de Strasbourg, F-67200 Strasbourg, France; cyril.bour@unistra.fr (C.B.); radhiabenbrika@gmail.com (R.B.); groh.audrey@orange.fr (A.G.); erwan.pencreach@chru-strasbourg.fr (E.P.); Eric.GUERIN@chru-strasbourg.fr (E.G.); guenot@unistra.fr (D.G.); 2 Laboratoire de Biochimie et Biologie Moléculaire, Hôpitaux Universitaires de Strasbourg, 67200 Strasbourg, France; 3 Laboratory of Bioimagery and Pathologies, UMR7021 CNRS, University of Strasbourg, 67200 Strasbourg, France; 4 Fédération de Médecine Translationnelle, 67000 Strasbourg, France; 5 U1113 INSERM/Unistra, IRFAC – Interface de Recherche Fondamentale et Appliquée en Cancérologie, F-67200 Strasbourg, France

**Keywords:** mTOR, Irinotecan, AZD2014, colon cancer, metastasis, orthotopic xenograft

## Abstract

Advanced colorectal cancer has a poor prognosis because of metastasis formation and resistance to combined therapies. Downstream of PI3K/Akt and Ras/MAPK pathways, the mTOR kinase plays a decisive role in treatment failure. We previously established that irinotecan has antiangiogenic properties and it is known that new mammalian target of rapamycin (mTOR) catalytic AZD inhibitors, unlike rapamycin, target both mTORC1 and mTORC2. Thus, we hypothesized that the complete inhibition of the PI3K/AKT/mTOR/HIF-1α axis with mTOR catalytic inhibitors and low doses of irinotecan may have antitumor effects. We showed that the AZD8055 and AZD2014 inhibitors were much more potent than rapamycin to reduce cell viability of four colon cell lines. On the other hand, whereas AZD2014 alone inhibits migration by 40%, the drug combination led to 70% inhibition. Similarly, neither irinotecan nor AZD2014 significantly reduced cell invasion, whereas a combination of the two inhibits invasion by 70%. In vivo, irinotecan and AZD2014 combination drastically reduced ectopic patient-derived colon tumor growth and this combination was more potent than Folfox or Folfiri. Finally, the combination totally inhibited liver and lung metastases developed from orthotopic implantation of SW480 cells. Thus, the use of mTOR catalytic inhibitors, in association with other chemotherapeutic agents like irinotecan at low doses, is potentially a hope for colon cancer treatment.

## 1. Introduction

Colorectal cancer (CRC) remains the third most common cause of cancer-related deaths worldwide. Despite the growing arsenal of chemotherapeutic and targeted agents, overall five-year survival rate is less than 10% at the metastatic stage of the disease [1]. 

Activation of PI3K/Akt and Ras/Raf/MEK/MAPK pathways is commonly involved in sporadic colon cancer pathogenesis. Downstream of these two pathways, mammalian target of rapamycin (mTOR), a Ser/Thr protein kinase involved in the regulation of cell growth, proliferation, and survival, is directly activated by the PI3K/Akt pathway, or directly and indirectly by the Ras/MAPK pathway [2]. mTOR exists in two distinct functional complexes, mTORC1 and mTORC2, which control protein synthesis and survival respectively [3,4,5]. Since the discovery of the drug rapamycin, more has been learned about the involvement of the mTOR pathway in cell proliferation and tumorigenesis [6]. Over the last 15 years, there has been a great interest for mTOR inhibition using rapamycin and its chemical analogues, everolimus and temsirolimus, which target only the mTORC1 complex, for antitumor therapy. However, mTOR inhibition in cancer, especially as monotherapy, has limited benefits, increasing survival by just few months, and several studies demonstrated the occurrence of drug resistance [7,8,9,10]. As an example, colon and breast tumors taken from patients after four weeks of treatment with everolimus showed higher levels of activated AKT compared to untreated samples [11]. Renewed interest in targeting the mTOR pathway has evolved through the discovery that mTORC2 directly phosphorylates the Ser473 residue of the AKT kinase in several diseases [12], making mTORC2 a new therapeutic target. The positive regulation of AKT by mTORC2 implicates mTOR in acting both upstream and downstream of AKT [3,13]. Whereas rapamycin and analogues are allosteric mTOR inhibitors, the new generation of mTOR catalytic inhibitors are ATP analogues, which inhibit both mTORC1 and mTORC2 kinase activities [11,14,15]. Due to the dual inhibition of rapamycin-insensitive mTORC2 and rapamycin-sensitive mTORC1, these catalytic inhibitors are expected to be more potent in clinical use [14] and data have shown that dual inhibition of mTORC1 and mTORC2 may overcome rapamycin resistance [16].

In addition to this oncogenic pathway activation, CRC, like many other solid tumors, is characterized by a hypoxic microenvironment [17]. Tumor hypoxia appears to be strongly associated with malignant progression and resistance to therapy [17]. Multiple mechanisms could be involved in the hypoxia-induced resistance to chemotherapeutic agents, including activation of cell proliferation, decreased apoptosis and cytotoxicity of drugs, and tissue acidosis due to a high glycolytic rate [17]. At the molecular level, tumor cell adaptation to hypoxia is regulated in part by the PI3K/AKT/mTOR pathway and by the transcription factors HIF-1α and HIF-2α, whose protein expression and transcriptional activity are also partly regulated by mTOR [18]. 

Several drug combinations do associate irinotecan as a first-line treatment of metastatic CRC and, as shown in vitro, irinotecan antitumor activity results from double-stranded DNA damage caused by stabilization of the TOP1-DNA complex [19]. Nevertheless, our team has previously shown in vivo, in patient-derived xenografts (PDXs), that irinotecan administered at low doses has a cytotoxic or cytostatic effect without inducing genotoxic lesions, but rather by inhibiting the accumulation of HIF-1α protein and abolishing tumor vascularization [20]. 

Based on these observations, our working hypothesis is that the vertical and complete inhibition of the PI3K/AKT/mTOR/HIF-1α axis with mTOR catalytic inhibitors and low doses of irinotecan may have a significant antitumor effect by reducing cancer cell viability and migration, as well as risk of recurrence of metastases. In clinics, the reduction of irinotecan doses is of particular interest in view of its severe toxicity [21].

## 2. Results

### 2.1. Effects of mTORC1 and mTORC2 Inhibition on Colon Cancer Cell Viability

**Figure 1:**
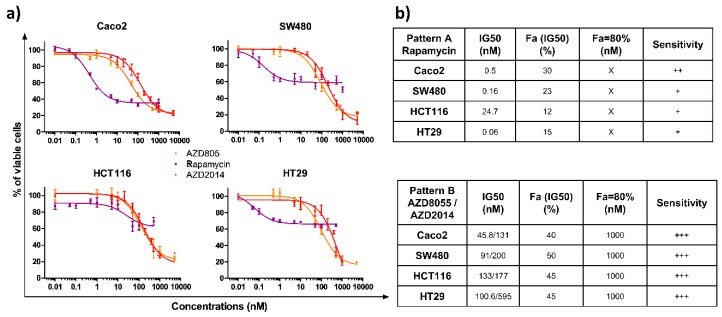
Effects of rapamycin, AZD8055 and AZD2014 on colon cancer cell viability. (**a**) The viability of colon cancer cells was determined by Crystal violet assay 72 hours after treatment. The four cell lines were treated with increasing concentrations of rapamycin, AZD8055, or AZD2014 (range of concentrations from 0.01 nM to 1000 nM for rapamycin and from 0,01 nM to 5000 nM for AZD). Each point corresponds to the mean ± SEM of three independent experiments, each carried out in quintuplicate. (**b**) The tables summarize the drug concentrations that inhibit the cell survival by 50% (IC50), the affected fraction (Fa) for a concentration equivalent to the IC50, the concentration required for an affected fraction of 80%, and sensitivity, which is estimated from + (low sensitivity) to +++ (high sensitivity). X denotes that an affected fraction of 80% could not be reached.

We evaluated the impact of the allosteric (rapamycin) and two catalytic mTOR inhibitors (AZD8055, AZD2014) on the survival of four colon cancer cell lines, characterized by specific mutations, leading to activated Ras/MAPK and PI3K signaling pathways (Table S1). Cells were treated with increasing doses of rapamycin (0.01 to 1000 nM), AZD8055, or AZD2014 (0.01 to 5000 nM) for 72 h and cell viability was quantified. The three compounds induced a dose-dependent growth inhibition of the four cell lines (Figure 1a). Interestingly, two patterns of inhibition were found depending on which mTOR inhibitor was tested (Figure 1b). The first pattern of inhibition, induced by rapamycin (Pattern A), is characterized by a differential sensitivity for each cell line. Indeed, the drug concentrations reducing the cell survival by 50% IC_50_ range from 0.06 for HT29 to 24.7 nM for HCT116, with a global moderate growth inhibition, with a maximal inhibition of 60% for the Caco2 cell line and 40% for the three other cell lines. In addition, increasing the rapamycin concentration above 50 nM did not further inhibit growth. The second pattern of inhibition (Pattern B) was observed with the two catalytic inhibitors (AZD8055 and AZD2014), which displayed a sensitivity range similar for all cell lines (Figure 1a). The IC_50_ concentrations were <150 nM with AZD8055 (IC_50_ ranging from 45.8 to 133 nM) and <500 nM with AZD2014 (IC_50_ ranging from 131 to 595 nM) for all cell lines. At 1000 nM, AZD8055 and AZD2014 cell survival was ≤20% (Figure 1b). 

### 2.2. Effects of mTORC1 and mTORC2 Inhibitors on mTOR Targets

**Figure 2:**
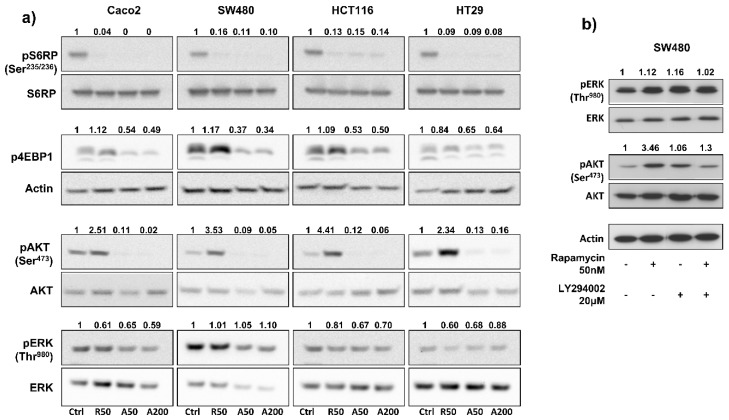
Benefits of AZD8055 and AZD2014 over rapamycin to inhibit PI3K/Akt/mTOR pathway. (**a**) Effects of treatment on phosphorylation of two main mTORC1 (S6RP and 4EBP1) and mTORC2 (AKT) targets, and of ERK after 24 hours treatment. WB are representative of 3 independent experiments. Ctrl: untreated cells; R50: rapamycin at 50 nM; A50: AZD8055 at 50 nM; A200: AZD2014 at 200 nM. (**b**) Analysis of ERK and AKT phosphorylation after 24h treatment with rapamycin (50 nM), LY294002 PI3K inhibitor (20 μM), or combination of both drugs in SW480 cells. Numbers represent quantification of the density ratio between phosphorylated and either non-phosphorylated form or actin, and normalized to the untreated cells.

To investigate the impact of drugs on blocking mTOR signaling, we measured, by Western blot, the expression of phospho-S6RP^Ser235/236^ (an mTORC1 effector) in colon cancer cell lines treated for 24 h with rapamycin, AZD2085, and AZD2014, at concentrations ranging from 50 to 500 nM. Results showed that concentrations of 50 nM for rapamycin, 50 nM for AZD8055, and 200 nM for AZD2014 almost completely abolished S6RP phosphorylation in each cell line (Figure S1); increasing drug concentrations didn’t bring any further benefit. Therefore, concentrations of 50 nM for rapamycin and AZD2085, and 200 nM for AZD2014 were used in the following in vitro experiments. We next evaluated the effects of mTOR inhibitors on the PI3K/AKT/mTOR pathway. Treatment of cells for 24 hours with AZD8055 and AZD2014, at 50 nM and 200 nM respectively, abolished phosphorylation of S6RP^Ser235/236^ and significantly inhibited phosphorylation of 4EBP1 (from 35% for HT29 to 63 % for SW480), two main mTORC1 effectors (Figure 2a). In contrast, rapamycin (50 nM) completely inhibited phosphorylation of S6RP^Ser235/236^, but didn’t have any effect on 4EBP1 phosphorylation. Interestingly, mTOR catalytic inhibitors also dramatically suppressed phosphorylation of AKT^Ser473^ (an mTORC2 effector), whereas in contrast, rapamycin induced phosphorylation of AKT^Ser473^ (from 2.3- to 4.4-fold increase depending on cell line; Figure 2a). Rapamycin and mTOR catalytic inhibitors had no significant effect on ERK phosphorylation (Figure 2a).

Interestingly, in SW480 cells, addition of a PI3K inhibitor (LY294002, 20 µM) to rapamycin (50 nM) prevented the 3.5-fold rapamycin-induced AKT phosphorylation (Figure 2b). This result suggests that rapamycin-induced phosphorylation of AKT depends on PI3K. 

In summary, AZD8055 and AZD2014 catalytic inhibitors are more effective than rapamycin in inhibiting the viability of colon cancer cells, and their efficacy is identical regardless of tumor cell mutational profile. In addition, rapamycin only partially inhibits mTORC1 pathway and leads to reactivation of PI3K/AKT pathway secondary to mTORC1 inhibition.

### 2.3. In Vitro Synergetic Activity of mTOR Inhibitors and Irinotecan on Colon Cancer Cells 

**Figure 3:**
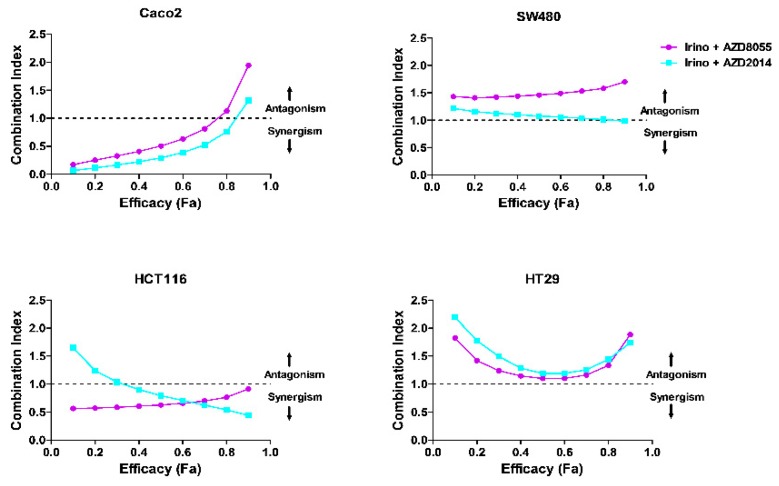
Synergism between irinotecan (Irino) and AZD8055, and between irinotecan (Irino) and AZD2014 on colon cancer cell viability. Combination index (CI) showing the synergistic effect of irinotecan (0.5 µM to 20 µM), AZD8055, or AZD2014 (25 nM to 1000 nM) on colon cancer cells. CI was determined from Chou and Talalay method, according to a constant ratio model. For a given efficacy (affected fraction, Fa), the combination index (CI) defines whether the association is synergistic (CI <1), additive (CI = 1) or antagonist (CI >1).

Based on the aforementioned in vitro results showing the better efficacy of catalytic inhibitors over rapamycin to inhibit viability of colon cancer cells when used alone, we next evaluated the effect of combining irinotecan (0.5 µM to 20 µM) or its active metabolite SN-38 (5 nM to 100 nM) with AZD8055 (5 nM to 500 nM) or AZD2014 (5 nM to 500 nM) on tumor cell viability. The protocol to establish drug combinations was designed according to the method reported by Chou and Talalay in order to quantify dose–effect relationships through calculation of a combination index (CI) [22,23]. Combination index values indicated a synergism between irinotecan and AZD8055 and between irinotecan and AZD2014 to inhibit cell viability in Caco2 and HCT116 cells (CI <1), and a slight additivity for the same combinations in HT29 cells (CI ~ 1, for an efficacy of 40% to 60%; Figure 3). In SW480 cells, the irinotecan + AZD8055 combination didn’t bring any benefit for inhibiting cell viability, CI value being above 1 (CI = 1.58), whereas the irinotecan + AZD2014 combination was additive (CI close to 1, Figure 3). When cells were treated with the active metabolite of irinotecan, SN38, the synergism between SN38 and both AZD was comparable to that observed for irinotecan and both AZD, for all cell lines (Figure S2). 

### 2.4. Effects of Treatments on PDX Growth 

Based on these results, we next determined the effect of the mTOR inhibitor AZD2014 alone or in combination with irinotecan on tumor growth in vivo. The PDX 36M1 was derived from a synchronous liver metastasis of a stage IV colon tumor, and the PDX 40 from a stage IV colon tumor, which presented two activating mutations in PIK3CA and ERBB2 genes (Tables S1 and S2). In vivo, we only evaluated the effect of the AZD2014 inhibitor, since AZD8055 has a strong hepatic turnover and will no longer be used in clinics [24]. In addition, only AZD2014 (vistusertib®) is currently under phase 2 clinical trials.

**Figure 4:**
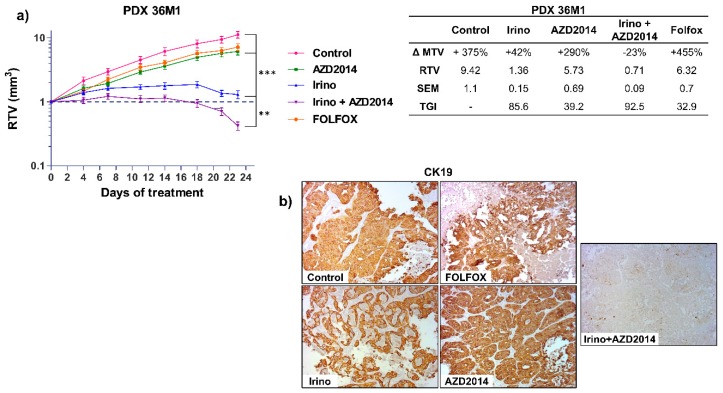
In vivo effect of irinotecan (Irino) and AZD2014 combination on tumor growth of PDX 36M1. (**a**) Relative tumor volume (mean RTV ± standard deviation) as a function of time for the PDX36M1. Each group consisted of 7 subcutaneous xenografted mice. The table summarizes the ΔMTV, RTV, and TGI for each group between the beginning and the end of treatment. ΔMTV (%): variation of the mean tumor volume between day 1 and the end of the treatments. RTV: Relative tumor volume. SEM: Standard error of mean. TGI (%): Tumor growth inhibition. (**b**) Immunostaining of PDX 36M1 tissue sections for CK19 at the end of treatments. The images are representative of all tumors from each group (magnification x100). The treatment schedules were as follows: irinotecan (Irino) (IP, 10 mg/kg/day q5d); AZD2014 (oral gavage, 20 mg/kg BID, 2 days on/5 days off); FOLFOX (5FU: 50 mg/kg, q7d, IP, LV: 90 mg/kg, q7d, IP, oxaliplatin: 6 mg/kg, q7d, IP). Control mice received no treatment. ** *p* < 0.005, *** *p* < 0.0005.

For the PDX 36M1, AZD2014 alone led to a slight reduction of tumor growth (RTV AZD2014 = 5.73; TGI = 39.2%; *p* = 0.03) whereas a combination of irinotecan + AZD2014 induced a regression of the tumor volume (RTV Irino + AZD2014 = 0.71; TGI: 92.5%; *p* < 0.0005) (Figure 4a). We next investigated whether this combination would have a better effect compared to the reference standard treatments for advanced colon cancer. Interestingly, the patient whose liver metastasis was used to derive PDX 36M1 progressed under treatment with FOLFOX, with progression of the disease six months after surgery (Table S2). Similarly, in nude mice, after 21 days of treatment, FOLFOX only slightly inhibited tumor growth (RTV FOLFOX = 6.32, TGI = 32.9%, *p* = 0.06), whereas as mentioned above, a combination of irinotecan + AZD2014 induced a cytotoxic effect with a TGI of 92.5% (Figure 4a). At the end of the treatment, histological analysis of the tumors treated with the irinotecan + AZD2014 combination revealed an important necrosis and very few cells (<5%) expressing the epithelial colon cell marker, cytokeratin 19 (CK19) (Figure 4b; Figure S3). These observations confirm the benefit of this combination over conventional therapeutic strategies. 

**Figure 5:**
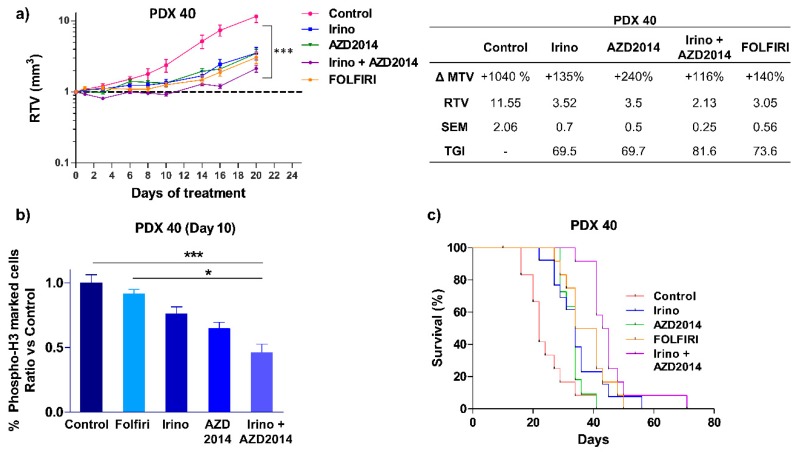
In vivo effect of irinotecan (Irino) and AZD2014 combination on PDX 40 tumor growth. (**a**) Relative tumor volume (mean RTV ± standard deviation) as a function of time for the PDX 40. Each group consisted of 7 subcutaneous xenografted mice. The table summarizes the ΔMTV, RTV and TGI for each group between the beginning and the end of treatment. ΔMTV (%): variation of the mean tumor volume between day 1 and the end of the treatments. RTV: Relative tumor volume. SEM: Standard error of mean. TGI (%): Tumor growth inhibition. (**b**) Quantification of cells positive for phospho-histone H3 in PDX 40, 10 days after initiation of treatment. Data are expressed as a relative ratio versus the control group. (**c**) Kaplan-Meier analysis of mouse survival for PDX 40 in each group of treatment. An event corresponds to the ethical sacrifice of the mouse due to a tumor volume reaching 1500 mm^3^ or because of tumor ulceration. The treatment schedules are as follows: irinotecan (Irino) (IP, 10 mg/kg/day q5d); AZD2014 (Oral gavage, 20 mg/kg BID, 2 days on/5 days off); FOLFIRI (5FU: 50 mg/kg, q7d, IP, LV: 90 mg/kg, q7d, IP, irinotecan 10 mg/kg, q5d, IP). Control mice received no treatment. * *p* < 0.05, *** *p* < 0.0005.

The same combination was tested on another PDX (PDX 40), which was derived from a stage IV colon tumor (Table S2) that escaped both FOLFOX and FOLFIRI regimens six months after surgery, leading to the death of the patient. The patient’s primary tumor and the derived PDX had activating PIK3CA E545K and ERBB2 L755S mutations (Table S1) and we previously reported that this PDX is resistant to irinotecan alone [20]. The present results showed that irinotecan + AZD2014 combination induced a cytostatic effect up to 12 days of treatment (RTV Irino + AZD2014 = 1.05), and thereafter by day 14, the tumor escaped with a recovery of tumor growth (Figure 5a). Nevertheless, at the end of the treatment (day 20), the observed TGI was higher when irinotecan was combined with AZD2014 (TGI Irino + AZD2014 = 81.6%, *p* < 0.0001) than when drugs were used alone (TGI Irino = 69.5%, *p* = 0.0007 and TGI AZD2014 = 69.7%, *p* = 0.0003), and also greater than that induced by FOLFIRI (TGI FOLFIRI = 73.6%, *p* = 0.0002, Figure 5a). 

Interestingly, tissue immunostaining with the proliferation marker phospho-histone H3^Ser10^ performed at day 10, showed that irinotecan + AZD2014 significantly decreased the percentage of proliferating cells as compared to control (*p* < 0.0005) or FOLFIRI (*p* < 0,05) (Figure 5b).

When considering mouse survival, which corresponds to the time at which mice were ethically sacrificed when tumor volume reached 1500 mm^3^, mice from the irinotecan + AZD2014 group had a significantly longer survival than mice treated with irinotecan alone (HR = 2.991; (1.176 – 7.606); *p* = 0.02) or AZD2014 alone (HR = 16.01, (4.399 – 58.27), *p* < 0.0001) (Figure 5c), and, importantly, mice from FOLFIRI group had shorter survival compared to mice treated with irinotecan + AZD2014 (HR = 2.474, (0.921 – 6.643), *p* = 0.07) (Figure 5c).

Overall, the treatments alone or in combination did not produce toxicity, as the mice weight followed a normal evolution during the period of treatment (Figure S4).

Taken together, our data confirm that combining irinotecan to AZD2014 is effective in inhibiting tumor growth of two metastatic PDX regardless of their mutational profile. In addition, this combination is more effective than the two standard regimens, FOLFIRI and FOLFOX, for the two PDX.

### 2.5. In Vitro Effects on Cell Migration and Invasion

Due to the fact that most of the patients with CRC die from metastases, we next investigated the inhibitory effects of irinotecan + AZD2014 combined treatment on the metastatic process. 

We first evaluated the in vitro effect of the drug combination on colon cancer cell migration and invasion after 24 h treatment. Impact of the treatment was evaluated only on SW480 cells, because the other cell lines did not sufficiently migrate in the Boyden chamber assay. Irinotecan (1 µM) did not significantly inhibit cell migration, whereas AZD2014 (200 nM) decreased migration by 40% as compared to untreated cells (*p* < 0.0005; Figure S4). The inhibitory effect increased up to 70% when AZD2014 was combined with irinotecan (irinotecan + AZD2014 vs. AZD2014: *p* < 0.05, Figure S5). Cell invasion capacity, evaluated after coating Boyden chambers with Matrigel, was not significantly decreased by AZD2014 (40% decrease) or irinotecan alone (4% decrease), but irinotecan + AZD2014 induced a significant inhibition of cell invasion (70% inhibition, *p* < 0.05 vs. untreated cells; Figure S5). Taken together, these results demonstrate that AZD2014 alone inhibited the migratory and invasive capacity of SW480 cells and that combining AZD2014 with irinotecan accentuated this effect.

### 2.6. In Vivo Effects on Metastasis Formation

**Figure 6:**
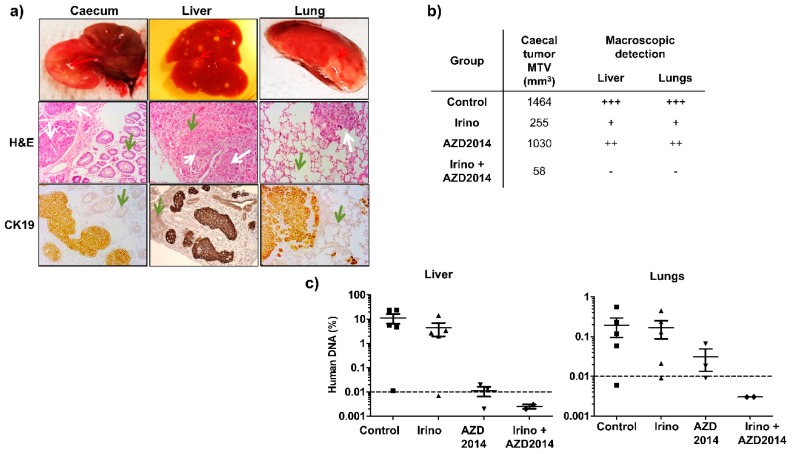
Effect of irinotecan and AZD2014 on in vivo metastasis 25 days after SW480 cell injections. (**a**) Caecum, liver and lungs of the orthotopically xenografted mice were removed and subjected to H&E and CK19 staining. Red arrows: macroscopic caecal tumor and spots of metastasis. White arrows: colon tumor cells, green arrows: normal tissue (caecal mucosa, liver, and lung) (magnification 200x). (**b**) Mean tumor volumes (MTV) of the caecal tumor and macroscopic detection liver and lung metastases (spots of necrosis). +++: numerous spots, ++: moderate spots, +: few spots, no spot. (**c**) Human DNA content in the liver and lungs of orthotopically xenografted mice at the end of treatment. The amount of human DNA in the organs was quantified by q-PCR of *Alu* sequences and expressed as a percentage of DNA input used for PCR amplification. Control: untreated mice, irinotecan (10 mg/kg, q5d, IP), AZD2014 (20 mg/kg, BID 2 days on/5 days off, oral gavage), irinotecan (10 mg/kg, q5d, IP) + AZD2014 (20 mg/kg, BID 2 days on/5 days off, oral gavage). Dashed line corresponds to the detection limit of the technique.

To confirm that irinotecan combined with AZD2014 can slow down invasion and metastasis formation in vivo, we developed orthotopic xenografts from SW480 cells (Figure 6a). To validate the capacity of the treatments to block the metastasis development, we used a validation group composed of two mice. These mice were sacrificed on the day of initiation of the treatments, i.e., seven days after the injection of the cell suspension, and their organs were collected. Detection of Alu sequences by quantitative PCR allowed us to confirm the presence of a caecum tumor, but no liver and pulmonary micrometastasis, before the initiation of treatments. 

At the time of sacrifice (day 25), all mice of the control group had developed a primary caecum tumor (Figure 6b). Concerning treated mice, at the end of the treatment (day 25), all the mice treated with AZD2014 had also developed a caecum tumor, whereas only 60% and 40% of the mice had a palpable tumor mass in the caecum wall for the irinotecan and irinotecan + AZD2014-treated groups, respectively. Moreover, the size of the primary tumor was decreased for mice treated with irinotecan, AZD2014, and the combination, as compared to the control group (Figure 6b). At day 25, macroscopic hepatic and pulmonary metastases were identified as spots of tissue necrosis on the organs (Figure 6a). By visual inspection of these organs, mice treated with the irinotecan + AZD2014 combination did not develop macroscopic metastases (Figure 6b).

In order to evidence or confirm the presence of microscopic and/or macroscopic metastases in the liver and lungs, we searched for human Alu sequences using quantitative PCR at the day of sacrifice (day 25). A standard curve established using various amounts of human DNA extracted from SW480 cells allowed us to correlate the cycle threshold (Ct) values for detection of Alu sequences and the amount of human DNA in the tissue. In the control group, 80% (four out of five) of mice developed hepatic and pulmonary metastases (Figure 6c). The average Alu sequences content was 14% and 0.2% for liver and lungs, respectively (Figure 6c). Mice treated with AZD2014 or irinotecan alone contained less Alu sequences in their organs as compared to the control group, with a respective rate of 4.4% and 0.015% in the liver, and 0.16% and 0.03% in the lungs. When mice were treated with the irinotecan + AZD2014 combination, no Alu sequence could be specifically amplified in their liver and lungs, suggesting very few or even no colon tumor cells in these organs (abundance of 0.03 pg of human DNA, corresponding to 0.003% invasiveness, below the detection limit of the technique) (Figure 6c).

## 3. Discussion

The present study aimed at demonstrating that complete inhibition of the PI3K/AKT/mTOR/HIF-1α axis with mTOR catalytic inhibitors and low doses of irinotecan significantly reduces cell viability and migration, as well as dissemination capacity. The presence of activating mutations at the level of the PI3K/AKT and Ras/MAPK pathways upstream of mTOR concerns 30% and 50% of the CRC cases, respectively [25]. An immunohistochemical study conducted on 154 patients, showed that colon tumors overexpress p-mTOR^Ser2448^ and p-p70S6K^Thr389^ [26]. Similarly, overexpression of mTOR mRNA is associated with decreased overall survival of patients with stage III CRC [27]. These data highlight the importance of targeting mTOR axis in CRC. At the present time, first and second generations of mTOR inhibitors are available. The first-generation allosteric inhibitors, which derive from rapamycin, inhibit the formation of the mTORC1 complex, and at the preclinical level, these rapalogs show limited efficacy in breast cancer and glioblastoma [28]. In particular, a biochemical approach has shown a reactivation of the PI3K/AKT pathway, secondary to the inhibition of mTORC1, by rapalogs [29].

Similarly, clinical studies using everolimus in CRC have shown modest activity, and combining everolimus and bevacizumab has a limited efficacy with an important toxicity [30,31]. This led to the development of second-generation catalytic inhibitors such as AZD8055 and its congener AZD2014, which inhibit the kinase activity of both mTORC1 and mTORC2 [32,33,34]. mTORC2 is one of the main activators of PI3K/AKT pathway and phosphorylates AKT^Ser473^ [13]. In the present study, we showed that catalytic mTOR inhibitors more effectively inhibited the viability of colon cancer cells than the allosteric inhibitor rapamycin, as rapamycin led to only a partial inhibition of the mTORC1 complex through a weak inhibition of 4EBP phosphorylation. In addition, rapamycin caused phosphorylation of AKT^Ser473^, which resulted from PI3K activation, secondary to mTORC1 inhibition. Conversely, the second-generation mTOR inhibitors completely inhibited the activity of the mTORC1 complex, as S6RP and 4EBP1 remained unphosphorylated and AKT^Ser473^ phosphorylation was almost completely abolished. These data are in full agreement with other studies [32,35]. Thus, in CRC, unlike first-generation inhibitors, mTOR catalytic inhibitors can completely block the mTOR axis. 

The rational use of therapeutic combinations is, today, the standard of care for patients. In our study, in vitro combination index highlights the benefit of combining irinotecan to mTOR catalytic inhibitors to inhibit cell proliferation. When using ectopic colon PDX characterized at the molecular level, and for their response to FOLFIRI or FOLFOX treatment, we further demonstrated in vivo the benefit of combining irinotecan to a catalytic mTOR inhibitor.

Our previous results showed that low-dose irinotecan results in a lack of tumor vascularization and inhibition of HIF-1α protein accumulation, leading to impaired adaptation of tumor cells to their environment [20]. We hypothesized that the efficacy of combinatorial synergism of the drugs relies on effects on tumor microenvironment such as vascularization, as well as on oncogenic pathways. Interestingly, Vétillard et al. have demonstrated in vitro and in vivo that SN38, the active metabolite of irinotecan, leads to a secondary activation of AKT in two colon cancer lines [36]. Thus, the inhibition of PI3K/AKT/mTOR with catalytic mTOR inhibitor could abolish this mechanism of resistance observed when using irinotecan. 

Development of targeted therapies requires the identification of biomarkers to predict tumor sensitivity, and there is currently no consensus on the biomarker(s) predicting responses to mTOR inhibitors alone or in combination. The use of PDX with different mutational profiles shows the efficacy of the combination on a colon tumor liver metastasis (PDX 36M1) and on a primary tumor mutated for PIK3CA and ERBB2 genes (PDX 40). These results suggest a potential benefit of low dose irinotecan combined with an mTOR catalytic inhibitor for tumors whose current therapeutic arsenal is limited. Indeed, tumors with KRAS or ERBB2 mutations have been shown to be resistant to anti-EGFR antibodies [37], whereas other studies have shown that tumors with mutations in PI3K pathway or loss of PTEN are more sensitive to mTOR inhibitors [38]. Moreover, we now know that colon cancer cells with a mutated TP53 gene have decreased sensitivity to irinotecan [39]. 

Our study also shows the benefit of the irinotecan + AZD2014 combination on migration and invasion in vitro and metastasis development in vivo. Indeed, mice orthotopically implanted with the SW480 cells and treated with the irinotecan + AZD2014 combination no longer developed liver and lung metastases, as evidenced by the expression of human Alu sequences. This can result from inhibition of PKCα activity and NFκB expression, as shown in glioblastoma [40]. In addition, use of shRNA directed against raptor or rictor leads to an increase in E-cadherin and a decrease in vimentin in vitro in the SW480 cells [41]. Inhibition of mTOR would, therefore, prevent epithelial-mesenchymal transition and inhibit tumor cell dissemination. 

## 4. Materials and Methods 

### 4.1. Cell Lines and Human Colon Tumor Samples

The HCT116 and SW480 colon cell lines were obtained from the American Type Culture Collection (ATCC, Manassas, VA, USA) in 2013. HT29 and Caco2 colon cell lines were kindly provided by Dr. M. Rousset [42]. For the 4 cell lines, the mutational status of *BRAF, PIK3CA, KRAS*, and 23 other genes was validated by next-generation sequencing (NGS) during 2018 (Tumor Hotspot MASTR Plus assay, Multiplicom-Agilent; Table S3). The 4 lines maintained the original mutational profile, indicating the absence of drift in the genomic anomaly profile of the cells. Every six months, the mycoplasma status was checked by PCR. They were all maintained in Dulbecco’s modified Eagle’s medium (DMEM) with 1 g/L glucose and supplemented with 10% of heat-inactivated fetal calf serum (FCS) at 37 °C in a humidified 5% CO_2_ atmosphere. 

Human colon tumor fragments used to generate PDX were obtained in accordance with the ethical standards of the institutional committee of the Hôpitaux Universitaires de Strasbourg and provided by the Centre de Ressources Biologiques (CRB). Clinical characteristics of the human colon tumors are indicated in Table S2.

### 4.2. Compounds

Irinotecan (Hospira, 20 mg/mL), 5-fluorouracil (5FU) (Accord, 50 mg/mL), oxaliplatin (Hospira, 5 mg/mL), and calcium folinate (leucovorin, LV) (Sandoz, 10 mg/mL) were provided by the Pharmacy Department of the Hôpitaux Universitaires de Strasbourg. Rapamycin was purchased from LC laboratories (R-5000), LY294002 from Calbiochem (440202), and AZD8055 from Selleckchem (S1555). The AZD2014 compound and the AZD8055 congener [24] were provided from AstraZeneca (Material Transfer Agreement, July 2015).

### 4.3. Molecular Profiling of PDX and Cell Lines by NGS

DNA was extracted from cultured cells and from snap frozen tissue of PDX using phenol/chloroform extraction protocol. Mutation screening was performed by NGS on a MiSeq Illumina platform using Tumor Hotspot MASTR Plus assay (Multiplicom-Agilent) (Table S3). Sequencing data were aligned to human genome hg19 using BWA-MEM algorithm (Burrows-Wheeler Aligner-Maximal Exact Matches). Variants were called using three different variant callers: VarScan, GATK HaplotypeCaller, and GATK UnifiedGenotyper. The minimum coverage per base and variant allelic frequency were fixed at 500-fold and 5% respectively. Data were visualized using the Integrative Genomics Viewer.

### 4.4. Cell Viability Assay

Cells were seeded in a 96-well plate overnight and treated with increasing concentrations of rapamycin (0.01 nM to 1000 nM), AZD8055, or AZD2014 (0.01 nM to 5000 nM) for 72 h. Cell viability was determined using crystal violet colorimetric assay. Calculation of the IC_50_ was performed with the GraphPad Prism5 software and corresponds to 50% of cell survival as compared to the maximal effect of the drugs.

### 4.5. Synergism Studies

Synergism was evaluated with irinotecan (0.5 µM to 20 µM), SN38 (5 nM to 100 nM), AZD8055 (5 nM to 500 nM), and AZD2014 (5 nM to 500 nM). The combination index was calculated according to Chou and Talalay [22,43] with CompuSyn1.0 software. 

### 4.6. Migration and Invasion Assays

Cell migration and invasion assays were performed using ThinCert™ 24-well cell culture Inserts for multiwell plates (8 μm pore, Greiner Bio-one). For invasion assay, the upper chamber of each multiwell insert (transwell) was precoated with Matrigel (250 μg/mL Corning Matrigel Matrix). Cells were pretreated for 24 h with irinotecan, AZD2014, or irinotecan + AZD2014. Four hours before seeding in the Boyden chamber, cells were maintained in serum-free DMEM before being loaded into the upper chamber of the transwell (40,000 viable cells/well as evidenced by trypan blue exclusion test, in a volume of 300 µL). After 24 h incubation, cells that had migrated to the lower chamber (containing 10% FCS) were fixed and stained with crystal violet. All experiments were performed in quadruplicate. Images were acquired using a Zeiss macroscope and the total number of cells that had migrated was quantified using Fiji software. For migration assay, the procedure was similar but without Matrigel coating.

### 4.7. Western Blot

After treatment, cells were lysed on ice in Laemmli buffer with protease and phosphatase inhibitor cocktails (Sigma, Saint-Quentin Fallavier, France). Nitrocellulose membranes were blocked in a 0.1% PBS-Tween with 4% nonfat dry milk and incubated overnight at 4 °C with primary antibodies specific for pS6RP^Ser235/236^ (1:1000, CST #2211), S6RP (1:1000, CST #2217), pAKT^Ser473^ (1:1000, CST #4060), AKT (1:1000, CST #4685), pERK^Thr202/Tyr204^ (1:1000, CST #4370), ERK (1:1000, CST #4695), and p4EBP1 (1:1000, CST #2855) from Cell Signaling (MA, USA) and actin (1:15000, MAB1501) from Millipore (CA, USA). Membranes were then incubated with horseradish peroxidase (HRP)-conjugated secondary antibodies (1:5000, NXA931 anti-mouse IgG or NA934V anti-rabbit IgG, GE Healthcare) followed by detection with the enhanced chemiluminescence kit (ECL Amersham, Velizy-Villacoublay, France). Signal intensity was quantified using Image J software (National Institutes of Health, Bethesda, MD, USA).

### 4.8. In Vivo PDX Experiments

All animal studies were conducted in accordance with the procedures outlined in the French Ethical Approval Apafis#16125-2018030716202418 v2 according to the European guidelines.

### 4.9. Ectopic Model

Human tumor tissues (PDX36M1 and PDX40) were transferred into DMEM supplemented with 200 UI/mL penicillin, 200 μg/mLstreptomycin, and 5 μg/mL fungizone (Gibco Thermofisher Scientific, Illkirch, France) and minced on ice. The resulting homogenate was injected subcutaneously into the right and left flanks of athymic 6–8 weeks old nu/nu nude mice (Janvier Labs, Le Genest Saint Isle, France) as previously described [44]. Mice were monitored twice a week and treatment started when tumors reached an average volume of 150–200 mm^3^. Mice were randomized according to tumor size into homogeneous groups of 7 mice. Irinotecan and rapamycin were given intraperitoneally at 10 mg/kg/day, q5d and 3 mg/kg/day, q5d, respectively. AZD2014 was given by oral gavage at 20 mg/kg, BID, 2 days on/5 days off [33]. FOLFOX protocol was as follows: 5FU: 50 mg/kg, q7d, IP; LV: 90 mg/kg, q7d, IP; oxaliplatin: 6 mg/kg, q7d, IP and FOLFIRI protocol was as follows: 5FU: 50 mg/kg, q7d, IP; LV: 90 mg/kg, q7d, IP; irinotecan: 10 mg/kg, q5d, IP [45,46,47]. Mean tumor volume (MTV), relative tumor volume (RTV, defined as the MTV ratio between a given day and the day of treatment initiation), and tumor growth inhibition (TGI = 100 − (RTVtreated/RTVcontrol) * 100) were calculated for each condition and compared between day 0 (before the initiation of the treatment) and the end of the treatment [20].

### 4.10. Orthotopic Model

NOD SCIDγ (NSG) 6–9-weeks-old mice (Charles River Laboratories, L’Arbresle, France) were used. Abdominal access by laparotomy was made through a 1 cm hypogastric midline skin and peritoneal wall incision. The caecum was exteriorized and placed on a sterile compress. The mucosa was pre-injured by a slight friction of the needle against the wall to facilitate implantation. This step is crucial to prevent leakage of tumor cells into the caecum lumen or peritoneal cavity. A 100 μl SW480 cell suspension (5 × 10^6^ cells) was injected into the caecum wall using a 30G needle. Seven days after implantation, mice were divided into 4 treatment groups (7 mice per group) and one validation group (2 mice). The mice of the validation group were sacrificed at the initiation of the treatments, 7 days after cell injection, and were used as a control for the presence of caecum tumor, liver, and pulmonary micrometastases. Treatment groups included control (untreated mice), irinotecan (irinotecan, 10 mg/kg, q5d, IP), AZD2014 (20 mg/kg, BID by oral gavage, 2 days on/5 days off), and irinotecan + AZD 2014-treated mice. Twenty-five days after implantation, control and treated mice were sacrificed and the caecum, liver, and lungs were collected. The organs of 2/7 mice were fixed in 4% paraformaldehyde for 4 hours for histological and immunohistochemistry or immunofluorescence analyses. The organs of the remaining 5/7 mice were snap-frozen in liquid nitrogen for subsequent molecular analyses (Figure S6).

### 4.11. Histology, Immunohistochemistry, and Immunofluorescence Analyses 

Resected fixed and paraffin-embedded tissues were processed for histology with H&E staining. For immunohistochemistry, 6 µm tissue sections were incubated with CK19 antibody (1:100, clone E16-L, DN103-05, Biotech DB, Kosice, Slovakia). For immunofluorescence, fixed sections were incubated with the primary phospho-histone H3^Ser10^ antibody (1:1000, 06–570, Millipore, St Quentin en Yvelines, France). Quantification of the number of pH3^Ser10^-labeled cells and the total number of cells were measured for two independent tumors per group (2 sections/tumor, 3 fields/section—objective 20x) using Fiji software.

### 4.12. Alu Sequence Quantification by Relative PCR

For Alu sequence quantification, liver and lungs of orthotopic xenografted mice were snap-frozen and stored at −80 °C. Whole frozen organs were transferred into ATL buffer (Qiagen, Courtaboeuf, France), mechanically dissociated (Kinematica Polytron PT 1300D), and incubated with proteinase K (1.87 mg/mL) overnight at 57 °C. Genomic DNA was extracted by the phenol/chloroform method. Quantitative relative PCR was performed on a LightCycler 480 (Roche, 38260 Meylan, France) with the QuantiTect SYBR Green PCR Master Mix® Kit (Qiagen). The primer used to detect the Alu sequence was as follows: forward sequence: 5’-CACCTGTAATCCCAGCACTTT-3’; reverse sequence: 5’-CCCAGGCTGGAGTGCAGT-3’. PCR reaction was carried out on 1 ng of genomic DNA in a final volume of 20 μl, combining SYBR Green I Master mix (1X final) with Hotstart Taq DNA polymerase, dNTPs, and 1 mmol/L MgCl_2_. The amount of human DNA present in the murine tissue was determined using a standard curve (0.01 pg to 1 ng of genomic DNA extracted from the human colonic cells SW480). Invasiveness was expressed by the amount of estimated human DNA in the total amount of DNA used for PCR reaction (1 ng). The detection limit of the technique, obtained by the analysis of liver and lungs of nonimplanted mice, was evaluated at 0.1 pg of human DNA (0.01% of the total analyzed DNA).

### 4.13. Statistical Analyses

All quantitative data are represented as mean ± SEM (standard error of mean). All statistical analyses were performed with GraphPad Prism 5 software (GraphPad Software, San Diego, CA, USA). Multigroup comparisons were made using a Kruskal–Wallis test, followed by a Dunn’s test. Comparisons of two groups were made using a Mann–Whitney test. One-sided decreasing Mann–Whitney tests were used to compare tumor volumes (efficacy) and log-rank tests were used to compare survival distributions (survival prolongation). *p*-values for tumor volume and survival assessment were adjusted for multiple comparisons according to Bonferroni–Holm. For all analyses, *p*-values under 0.05 represent statistically significant effect.

## 5. Conclusions

Overall, our data clearly and originally demonstrate the benefit of using drug combinations such as irinotecan and catalytic mTOR inhibitors to prevent tumor progression and/or the dissemination process. The use of mTOR inhibitors is potentially a hope in colon cancer treatment and the rational for association with other chemotherapeutic agents like irinotecan seems useful, but requires the identification of subgroups of patients that are most likely to respond. 

## Supplementary Materials

The following are available online at https://www.mdpi.com/2072-6694/11/10/1581/s1, Figure S1: Effects of rapamycin, AZD8055 and AZD2014 on mTOR pathway, Figure S2: Synergism between SN38 and AZD8055 and between SN38 and AZD2014 on colon cancer cell viability, Figure S3: In vivo effect of irinotecan (Irino) and AZD2014 combination on PDX 36M1, Figure S4: In vivo effect of irinotecan (Irino) and AZD2014 combination on mouse weight after PDX36M1 and PDX40 ectopic implantation, Figure S5: Effect of irinotecan and AZD2014 on SW480 cell migration and invasion, Figure S6: Effect of irinotecan and AZD2014 on in vivo metastasis, Table S1: Molecular characteristics of colon cell lines and PDX Table S2: Clinical parameters of human colon tumors used for patientderived xenografts (PDX), Table S3: List of genes and exons covered by the Tumor Hotspot MASTR Plus assay (Multiplicom/Agilent) used for NGS sequencing.
