# Supplementary material for: Synergistic Anti-Tumor Effect of mTOR Inhibitors with Irinotecan on Colon Cancer Cells
Source: Cancers (Basel). 2019 Oct 17;11(10):1581. doi: 10.3390/cancers11101581 (PMC6826690; doi:10.3390/cancers11101581)
Supplement: Supplementary file 1 [file cancers-11-01581-s001.zip › cancers-554916-SI/Supplementary Data/cancers-554916-Supplementary Tables .pdf]

Table S1. Molecular characteristics of colon cell lines and PDX.

Samples	*MSI/MSS*	*KRAS*	*HRAS*	*NRAS*	*BRAF*	*PIK3CA*	*PTEN*	*Others*
**Caco2**	MSS	WT	WT	WT	WT	WT	WT	WT
**SW480**	MSS	c.35G>T p.G12V	WT	WT	WT	WT	WT	WT
**HCT116**	MSI	c.38G>A p.G13D	WT	WT	WT	c.3140A>G p.H1047R	WT	WT
**HT29**	MSS	WT	WT	WT	c.1799T>A p.V600E	c.1345C>A p.P449T	WT	WT
**PDX 36M1**	MSS	WT	WT	WT	WT	WT	WT	WT
**PDX 40**	MSS	WT	WT	WT	WT	c.1633G>A p.E545K	WT	ERBB2 c.2264T>C p.L755S

Table S2. Clinical parameters of human colon tumors used for patient-derived xenografts (PDX).

	**PDX 36M1**	**PDX 40**
**TNM**	pT3N2M1	pT4N1M1
**Stage**	IV	IV
**Tumor location**	Synchronous hepatic metastasis of a left colon tumor	Right colon
**Metastasis localization**	Liver	Liver
**Sex**	M	F
**Age (years)**	74	77
**Treatment after surgery**	FOLFOX	FOLFOX/FOLFIRI
**Evolution**	Disease progression after 6 months. Death related to cancer 2 years after surgery	Disease progression at hepatic level. Death related to cancer 6 months after surgery

Table S3. List of genes and exons covered by the Tumor Hotspot MASTR Plus assay (Multiplicom/Agilent) used for NGS sequencing.

AKT1: NM_005163.2, exon 3
ALK: NM_004304.4, exons 20 to 29
BRAF: NM_004333.5, exons 11 and 15
CDKN2A: NM_000077.4 (p16/INK4a) and NM_058195.3 (p14/ARF), whole coding sequence (3 exons)
CTNNB1: NM_001904.3, exon 3
DDR2: NM_006182.3, whole coding sequence (16 exons)
EGFR: NM_005228.4, exons 18 to 21
ERBB2/HER2: NM_004448.3, exons 19, 20 and 21
ERBB4/HER4: NM_005235.2, exons 10 and 12
FGFR2: NM_000141.4, exons 7, 12 and 14
FGFR3: NM_000142.4, exons 7, 9, 14 and 16
H3F3A: NM_002107.4, exon 2
HIST1H3B: NM_003537.3, exon 1
HRAS: NM_005343.3, exons 2, 3 and 4
IDH1: NM_005896.3, exon 4
IDH2: NM_002168.3, exon 4
KIT: NM_000222.2, exons 8, 9, 10, 11, 13, 14, 17 and 18
KRAS: NM_033360.3, exons 2, 3 and 4
MAP2K1/MEK1: NM_002755.3, exons 2 and 3
MET: NM_001127500.2, exons 2, 10, 14 to 20
NRAS: NM_002524.4, exons 2, 3 and 4
PDGFRA: NM_006206.5, exons 12, 14 and 18
PIK3CA: NM_006218.3, exons 2, 3, 10, 11 and 21
PIK3R1: NM_181523.2, exons 11, 12 and 13
PTEN: NM_000314.6, whole coding sequence (9 exons)
STK11/LKB1: NM_000455.4, whole coding sequence (9 exons)
