# Supplementary material for: Synergistic Anti-Tumor Effect of mTOR Inhibitors with Irinotecan on Colon Cancer Cells
Source: Cancers (Basel). 2019 Oct 17;11(10):1581. doi: 10.3390/cancers11101581 (PMC6826690; doi:10.3390/cancers11101581)
Supplement: Supplementary file 1 [file cancers-11-01581-s001.zip › cancers-554916-SI/Supplementary Data/cnacers-554916-Supplementary Figures D. Reita.pptx]

## Slide 1
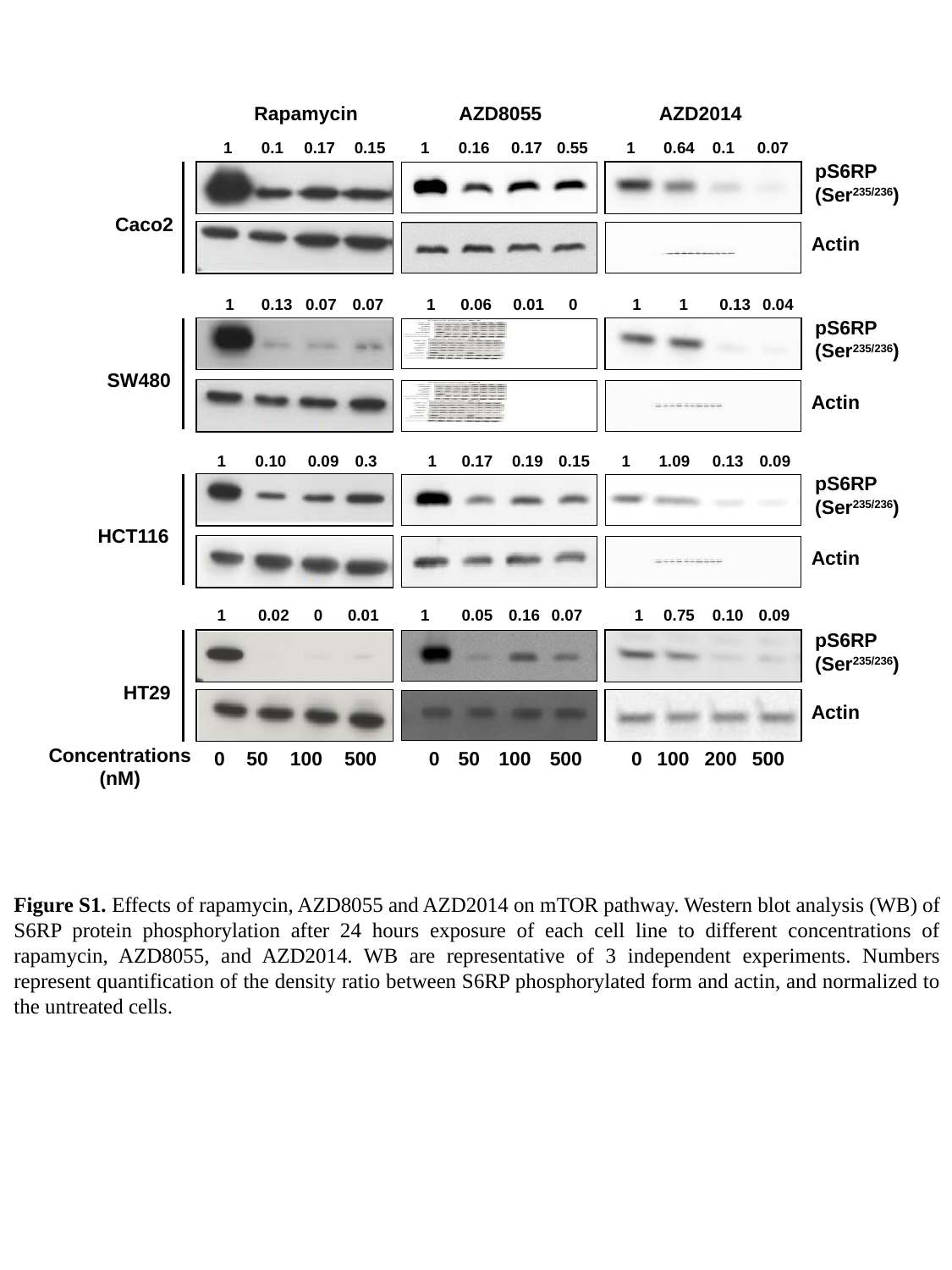

Rapamycin
AZD8055
AZD2014
1
0.1
0.17
0.15
1
0.16
0.17
0.55
1
0.64
0.1
0.07
pS6RP
(Ser235/236)
Caco2
Actin
1
0.13
0.07
0.07
1
0.06
0.01
0
1
1
0.13
0.04
pS6RP
(Ser235/236)
SW480
Actin
1
0.10
0.09
0.3
1
0.17
0.19
0.15
1
1.09
0.13
0.09
pS6RP
(Ser235/236)
HCT116
Actin
1
0.02
0
0.01
1
0.05
0.16
0.07
1
0.75
0.10
0.09
pS6RP
(Ser235/236)
HT29
Actin
Concentrations (nM)
0
50
100
500
0
50
100
500
0
100
200
500
Figure S1. Effects of rapamycin, AZD8055 and AZD2014 on mTOR pathway. Western blot analysis (WB) of S6RP protein phosphorylation after 24 hours exposure of each cell line to different concentrations of rapamycin, AZD8055, and AZD2014. WB are representative of 3 independent experiments. Numbers represent quantification of the density ratio between S6RP phosphorylated form and actin, and normalized to the untreated cells.

## Slide 2
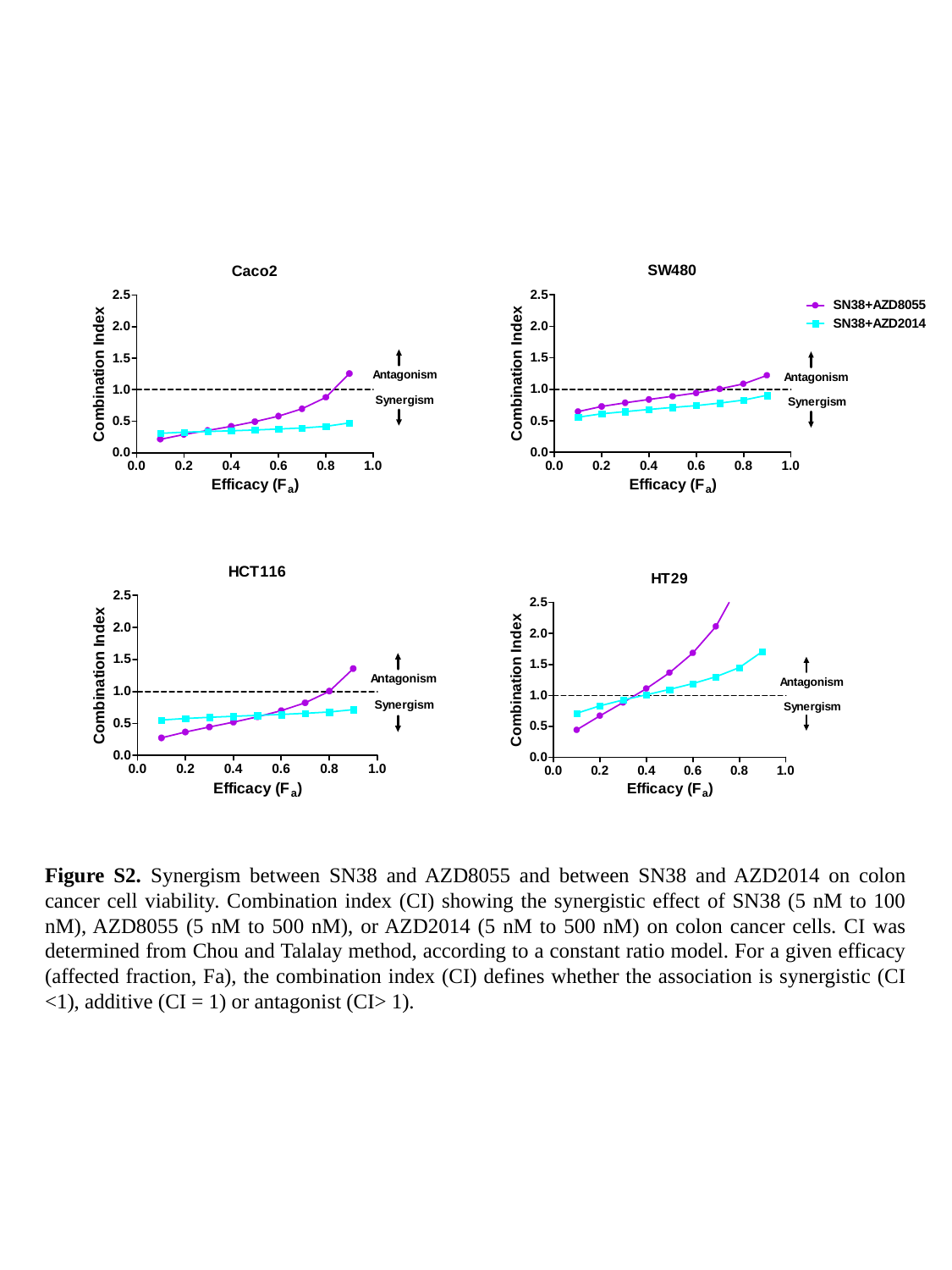

Figure S2. Synergism between SN38 and AZD8055 and between SN38 and AZD2014 on colon cancer cell viability. Combination index (CI) showing the synergistic effect of SN38 (5 nM to 100 nM), AZD8055 (5 nM to 500 nM), or AZD2014 (5 nM to 500 nM) on colon cancer cells. CI was determined from Chou and Talalay method, according to a constant ratio model. For a given efficacy (affected fraction, Fa), the combination index (CI) defines whether the association is synergistic (CI <1), additive (CI = 1) or antagonist (CI> 1).

## Slide 3
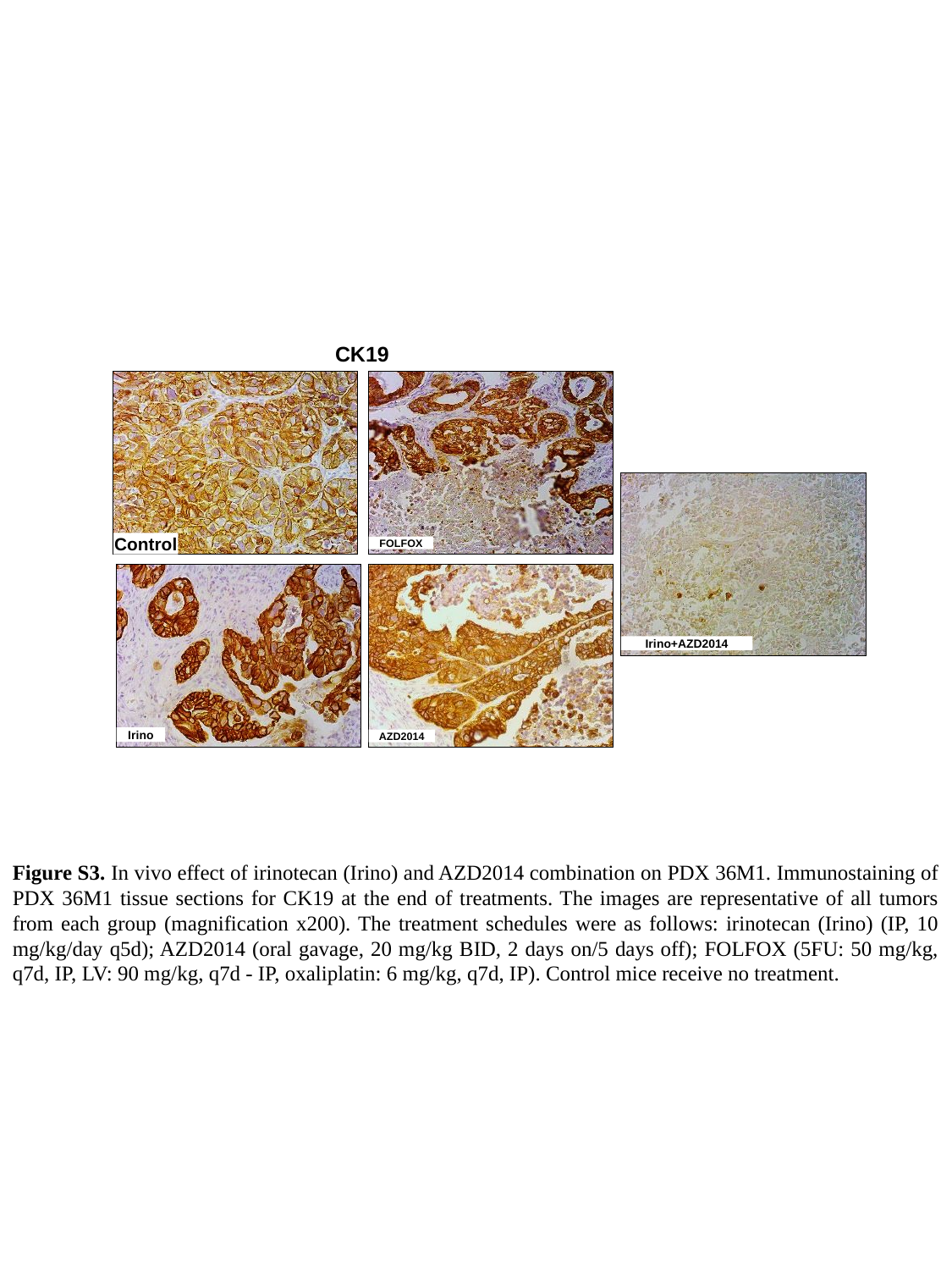

CK19
Control
FOLFOX
Irino+AZD2014
Irino
AZD2014
Figure S3. In vivo effect of irinotecan (Irino) and AZD2014 combination on PDX 36M1. Immunostaining of PDX 36M1 tissue sections for CK19 at the end of treatments. The images are representative of all tumors from each group (magnification x200). The treatment schedules were as follows: irinotecan (Irino) (IP, 10 mg/kg/day q5d); AZD2014 (oral gavage, 20 mg/kg BID, 2 days on/5 days off); FOLFOX (5FU: 50 mg/kg, q7d, IP, LV: 90 mg/kg, q7d - IP, oxaliplatin: 6 mg/kg, q7d, IP). Control mice receive no treatment.

## Slide 4
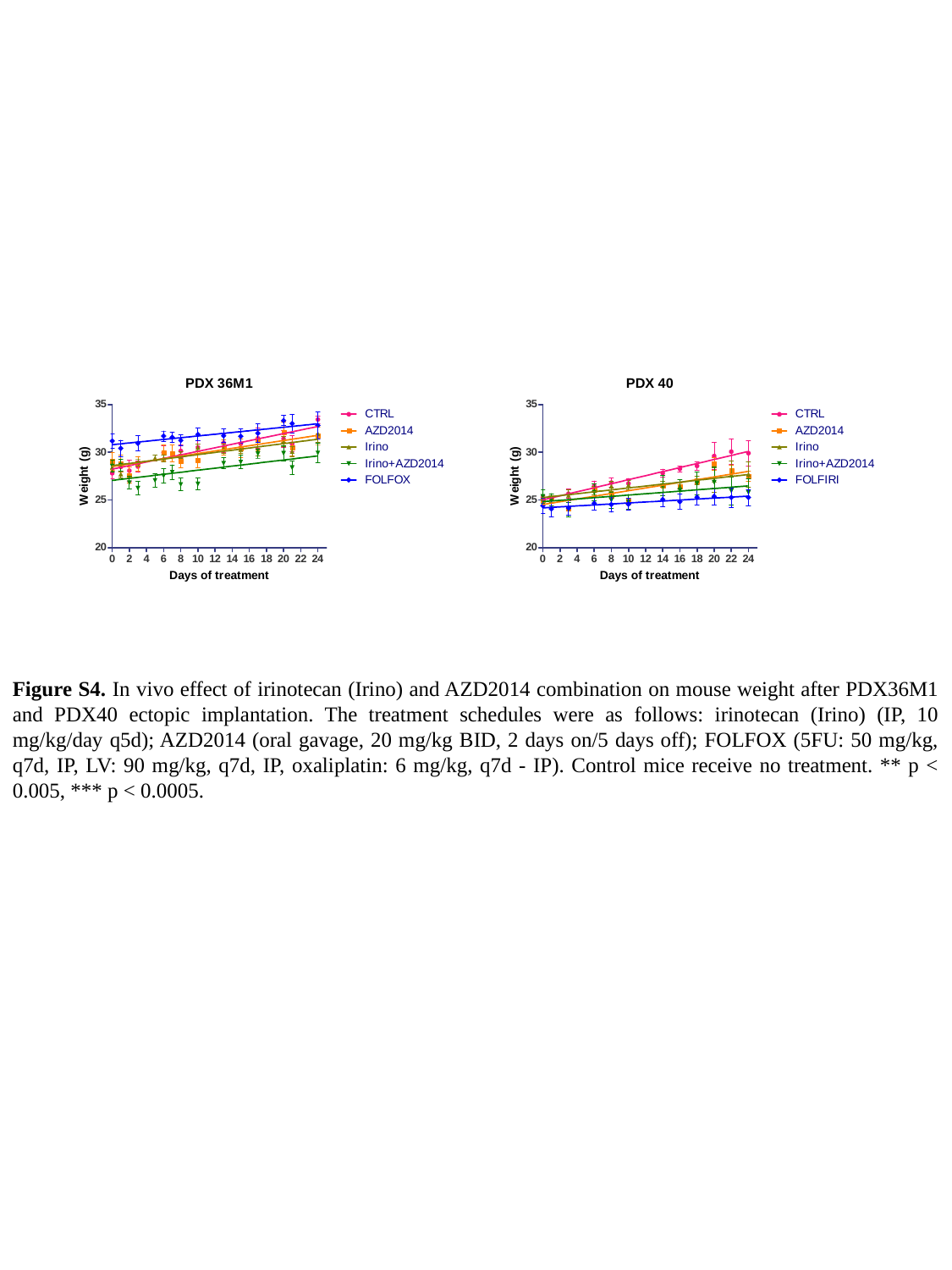

Figure S4. In vivo effect of irinotecan (Irino) and AZD2014 combination on mouse weight after PDX36M1 and PDX40 ectopic implantation. The treatment schedules were as follows: irinotecan (Irino) (IP, 10 mg/kg/day q5d); AZD2014 (oral gavage, 20 mg/kg BID, 2 days on/5 days off); FOLFOX (5FU: 50 mg/kg, q7d, IP, LV: 90 mg/kg, q7d, IP, oxaliplatin: 6 mg/kg, q7d - IP). Control mice receive no treatment. ** p < 0.005, *** p < 0.0005.

## Slide 5
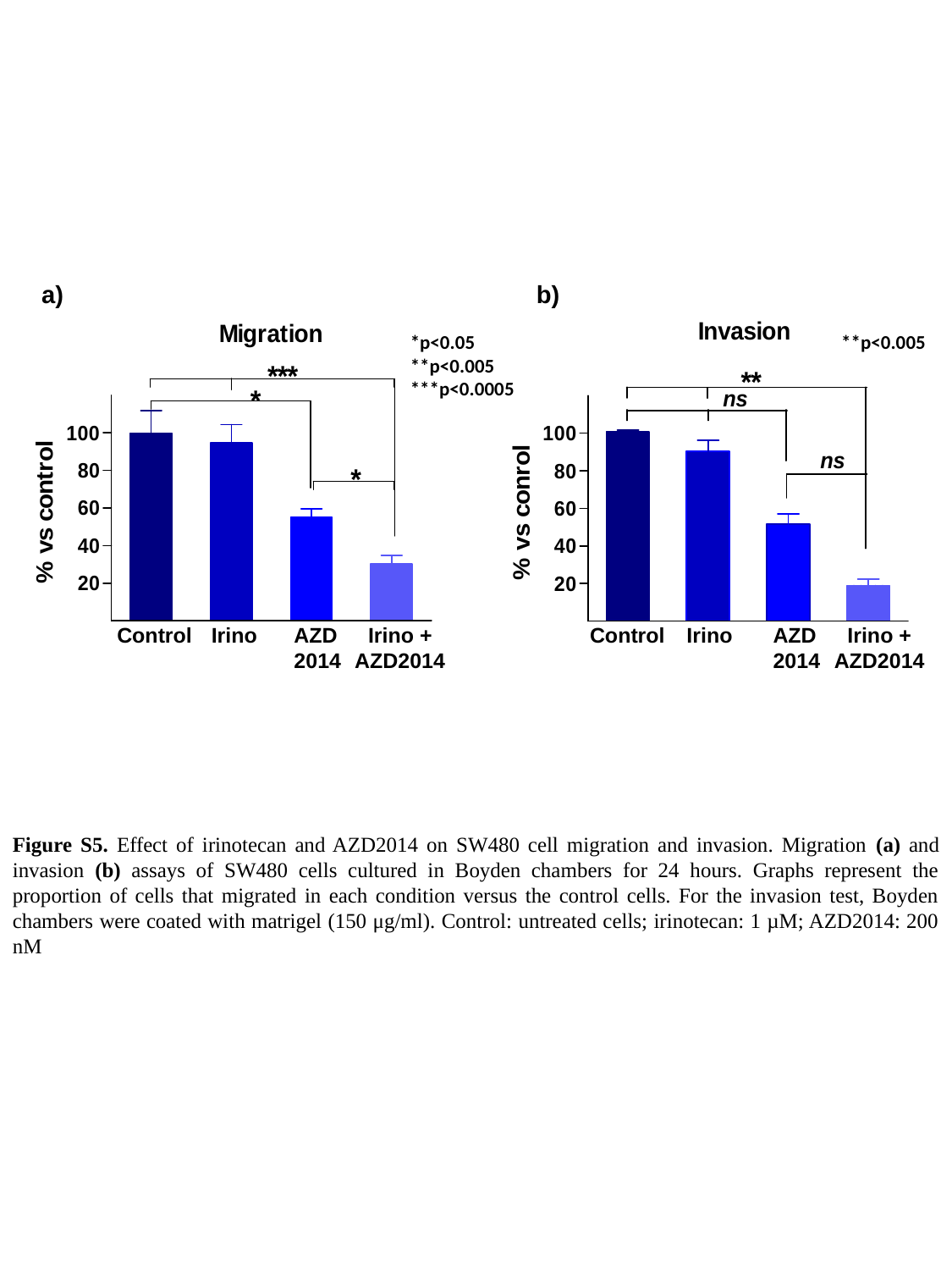

a)
b)
*p<0.05
**p<0.005
***p<0.0005
**p<0.005
Control
Irino
AZD
2014
Irino +
AZD2014
Control
Irino
AZD
2014
Irino +
AZD2014
Figure S5. Effect of irinotecan and AZD2014 on SW480 cell migration and invasion. Migration (a) and invasion (b) assays of SW480 cells cultured in Boyden chambers for 24 hours. Graphs represent the proportion of cells that migrated in each condition versus the control cells. For the invasion test, Boyden chambers were coated with matrigel (150 μg/ml). Control: untreated cells; irinotecan: 1 µM; AZD2014: 200 nM

## Slide 6
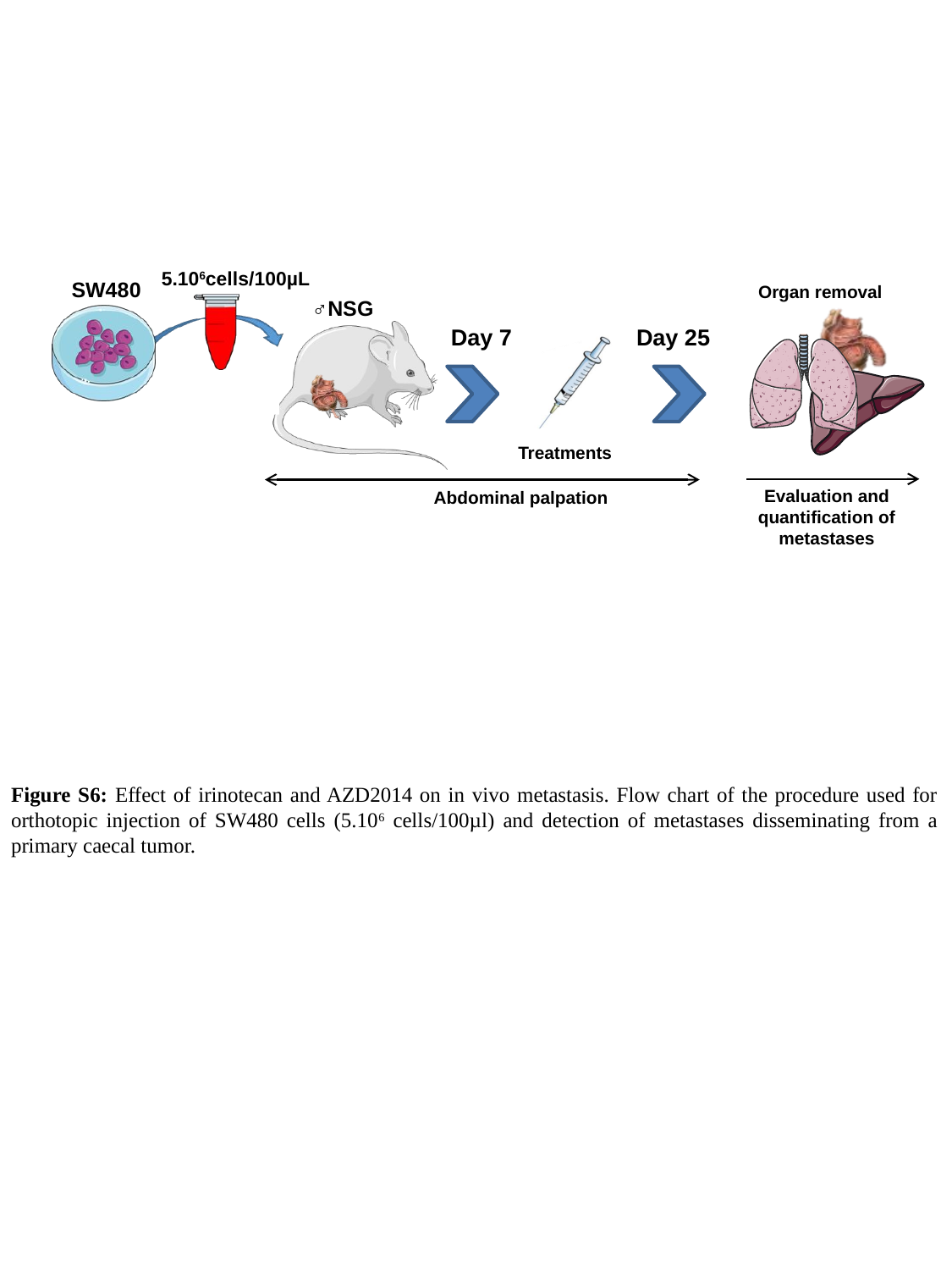

5.106cells/100µL
SW480
Organ removal
♂NSG
Day 7
Day 25
Treatments
Evaluation and quantification of metastases
Abdominal palpation
Figure S6: Effect of irinotecan and AZD2014 on in vivo metastasis. Flow chart of the procedure used for orthotopic injection of SW480 cells (5.106 cells/100µl) and detection of metastases disseminating from a primary caecal tumor.
